# Dynamics of skyrmion in disordered chiral magnet of thin film form

**DOI:** 10.1038/s41598-019-41441-5

**Published:** 2019-03-25

**Authors:** Wataru Koshibae, Naoto Nagaosa

**Affiliations:** 1 RIKEN Center for Emergent Matter Science (CEMS), Wako, Saitama, 351-0198 Japan; 2 Department of Applied Physics, The University of Tokyo, 7-3-1, Hongo, Bunkyo-ku, Tokyo, 113-8656 Japan

## Abstract

Magnetic skyrmion is a topological spin texture characterized by the mapping from the two dimensional real space to the unit sphere. It is realized in chiral magnets under an external magnetic field in the plane perpendicular to it. In thin film samples, which are most relevant to the applications, the thickness of the system parallel to the magnetic field is finite, and a skyrmion turns into a skyrmion string, which is often assumed to be a straight rod. There are phenomena related to the internal degrees of freedom along the string, e.g., the monopole and anti-monopole creation/annihilation, corresponding to the change in the skyrmion number. However, the role of this finite thickness in the topological stability and dynamics has not been explored yet. Here we study theoretically the current-driven dynamics of a skyrmion string under disorder potential by systematically changing the thickness of the sample to reveal the dynamical phase diagram in the plane of current density and thickness. We found the three regions, i.e., (i) pinned skyrmion string, (ii) moving depinned skyrmion string, and (iii) annihilation of skyrmion string, for thin and thick limits while (iii) is missing in the intermediate case. This indicates that there is the optimal range of thickness for the topological stability of skyrmion string enhanced compared with a two-dimensional skyrmion. This result provides a way to design and control skyrmions in thin films and interfaces of finite thickness.

## Introduction

Magnetic skyrmion^1–16^ is a two-dimensional topological spin texture characterized by an integer called skyrmion number which counts how many times the spin directions wrap the unit sphere. This topological nature leads to the variety of novel properties of skyrmions such as their topological stability, high mobility with very small threshold current density, and topological Hall effect. The crystal form of skyrmions has been first observed in three dimensional bulk crystal of chiral magnet MnSi by the neutron scattering experiment^6^, where the skyrmionic phase is limited to the small region in the plane of temperature and magnetic field. In three-dimensions, the skyrmions form the rod-like string along the direction of the external magnetic field. Later it has been recognized that the skyrmion phase is more stable in two-dimensional systems such as thin film by the real-space observation in terms of the Lorentz electron microscopy in (Fe,Co)Si^8^. In two-dimensions, a skyrmion is a point-like particle. From the viewpoint of applications, the proposals of skyrmionic devices have been discussed (e.g., refs ^17–19^), and interfaces or thin films^20–28^ offer an important laboratory since it naturally introduces the Dzyaloshinskii-Moriya (DM) interaction^29–31^ in a controllable way. However, the thickness of the sample is usually larger than the atomic lattice constant even in thin films and interfaces, and the degrees of freedom along the external magnetic field are still important. The distortion of the skyrmion string along the magnetic field is an important issue especially in the current-driven motion with impurities. The singular spin configurations associated with the creation and annihilation of skyrmions are called hedgehog or antihedgehog, corresponding to the monopole and antimonopole of emergent magnetic field, respectively^32–37^. Compared with the skyrmion dynamics in two-dimensions, the dynamics of skyrmion strings are less studied up to now especially during the current-driven motion. The mechanism for the creation of the monopole and antimonopole is an important issue, which is not easy to access experimentally but can be studied by numerical simulations.

In this paper, we investigate the current-driven dynamics of skyrmion with disorder by numerically solving the Landau-Lifshitz-Gilbert (LLG) equation for spins. To examine the essential role of the string degree of freedom of the skyrmion along the external magnetic field, we focus on the system thickness or string length dependence on its dynamics. By the numerical simulation of a skyrmion string under current, we find that the impurity effect strongly depends on the length of the skyrmion string. In particular, the theoretical results show that the impurity pinning is strongly suppressed by the unique dynamics of the skyrmion string. For a longer skyrmion string, we find its annihilation by the monopole-antimonopole creation through the excitation of the collective mode, i.e., breathing mode, along the string. These findings indicate that there exists an optimum condition for the stability of the current driven skyrmion which is of crucial importance not only from the viewpoint of fundamental physics on the topological magnetic texture but also applications.

## Results

### Model and Simulation

The model Hamiltonian used in this study is the following:

1 $$\begin{array}{ccc}{\mathscr{H}} & = & -\,J\,\sum _{{\boldsymbol{r}}}\,{{\boldsymbol{n}}}_{{\boldsymbol{r}}}\cdot ({{\boldsymbol{n}}}_{{\boldsymbol{r}}+\hat{{\boldsymbol{x}}}}+{{\boldsymbol{n}}}_{{\boldsymbol{r}}+\hat{{\boldsymbol{y}}}}+{{\boldsymbol{n}}}_{{\boldsymbol{r}}+\hat{{\boldsymbol{z}}}})+\,\sum _{{\boldsymbol{r}}}\,({{\boldsymbol{n}}}_{{\boldsymbol{r}}}\times {{\boldsymbol{n}}}_{{\boldsymbol{r}}+\hat{{\boldsymbol{x}}}}\cdot {{\boldsymbol{D}}}_{{\rm{X}}}+{{\boldsymbol{n}}}_{{\boldsymbol{r}}}\times {{\boldsymbol{n}}}_{{\boldsymbol{r}}+\hat{{\boldsymbol{y}}}}\cdot {{\boldsymbol{D}}}_{{\rm{Y}}}+\,{{\boldsymbol{n}}}_{{\boldsymbol{r}}}\times {{\boldsymbol{n}}}_{{\boldsymbol{r}}+\hat{{\boldsymbol{z}}}}\cdot {{\boldsymbol{D}}}_{{\rm{Z}}})-\,{K}_{{\rm{i}}{\rm{m}}{\rm{p}}}\,\sum _{{{\boldsymbol{r}}}_{i}\in {\rm{\Lambda }}}\,{({n}_{z,{{\boldsymbol{r}}}_{i}})}^{2}-h\,\sum _{{\boldsymbol{r}}}\,{n}_{z,{\boldsymbol{r}}},\end{array}$$

with $\hat{{\boldsymbol{x}}}$, $\hat{{\boldsymbol{y}}}$ and $\hat{{\boldsymbol{z}}}$ being the unit vectors in the *x*-, *y*- and *z*- directions, respectively. The normalized magnetic moment at site ${\boldsymbol{r}}=({r}_{x},{r}_{y},{r}_{z})$ is expressed by ***n***_***r***_. The lattice constant *a* is taken as the unit of length. The second term represents the antisymmetric DM interaction. We use ${{\boldsymbol{D}}}_{{\rm{X}}}=D\hat{{\boldsymbol{x}}}$, ${{\boldsymbol{D}}}_{{\rm{Y}}}=D\hat{{\boldsymbol{y}}}$ and ${{\boldsymbol{D}}}_{{\rm{Z}}}=D\hat{{\boldsymbol{z}}}$ for the model Hamiltonian of the chiral magnets, so that Bloch skyrmion dynamics is discussed. For the interface driven DM systems with thin film form, we use ${{\boldsymbol{D}}}_{{\rm{X}}}=-\,D\hat{{\boldsymbol{y}}}$, ${{\boldsymbol{D}}}_{{\rm{Y}}}=D\hat{{\boldsymbol{x}}}$ and ${{\boldsymbol{D}}}_{{\rm{Z}}}={\bf{0}}$, so that Néel skyrmion dynamics is discussed. In the case that ${{\boldsymbol{D}}}_{{\rm{Z}}}={\bf{0}}$, at least in this *minimal* model Hamiltonian, the dynamics of the magnetic texture is the same as that in the system with ${{\boldsymbol{D}}}_{{\rm{X}}}=D\hat{{\boldsymbol{x}}}$ and ${{\boldsymbol{D}}}_{{\rm{Y}}}=D\hat{{\boldsymbol{y}}}$. The single spin anisotropy *K*_imp_ is introduced on the random sites ${{\boldsymbol{r}}}_{{\rm{i}}}\in {\rm{\Lambda }}$ (${\rm{\Lambda }}$: set of the random sites), which represents the disorder effect. In reality, the impurity potential has finite spatial extent. To mimic such impurity potential profile, we introduce *dense* impurities where the impurity-impurity distance is much smaller than skyrmion size. For the skyrmion, therefore, the impurities act as rather a spatially slowly varying potential rather than the point-like one. In the system without the disorder, i.e., ${K}_{{\rm{imp}}}=0$, the competition between the exchange interaction *J* and DM interaction *D* results in the single-*q* helix state with $q=D/J$, for $h=0$. With increasing *h*, the skyrmion crystal state and the ferromagnetic state appears successively^38,39^. In this paper, we put ${{\boldsymbol{D}}}_{Z}=D\hat{{\boldsymbol{z}}}$ in most cases and we use the parameter set $\{J=1,D=0.2,h=0.6\}$ where the metastable skyrmion exists in the ferromagnetic background. For the impurities, we introduce ${K}_{{\rm{imp}}}=0.2$ with 10% concentration. (see also Methods).

The magnetic skyrmions discussed here is topologically the same as the magnetic bubble where the long-range magnetic dipolar interaction^40–43^ plays essential role. Usually, such skyrmionic state (or nomal magnetic bubble) has several hundreds nanometer to sub-micrometer scale size. In those cases, the twist of magnetic moment alignment between Bloch and Néel winding textures occurs along the thickness direction for the static skyrmionic states^40,41,44–46^. For such larger size magnetic skyrmion(s), however, different degree of freedom becomes important, i.e., the flexibility of the magnetic winding texture causes substantial deformation of the skyrmionic state during its dynamics. Those flexibilities induces complex skyrmion dynamics^42,43^. When the DM interaction dominates the winding magnetic texture, the skyrmion size becomes small, and it is typically several tens nanometer scale or smaller. In those cases, the magnetic bubble characteristics discussed above are reduced. Here, we discuss the limiting case, i.e., the DM interaction driven magnetic skyrmion and its dynamcs. Even in this simplified systems, skyrmion string shows rich dynamical behaviors.

The LLG equation is given by:

2 $$\begin{array}{ccc}\frac{{\rm{d}}{{\boldsymbol{n}}}_{{\boldsymbol{r}}}}{{\rm{d}}t} & = & -\,\gamma \frac{{\rm{\partial }}{\mathscr{H}}}{{\rm{\partial }}{{\boldsymbol{n}}}_{{\boldsymbol{r}}}}\times {{\boldsymbol{n}}}_{{\boldsymbol{r}}}+\alpha {{\boldsymbol{n}}}_{{\boldsymbol{r}}}\times \frac{{\rm{d}}{{\boldsymbol{n}}}_{{\boldsymbol{r}}}}{{\rm{d}}t}-\,({{\boldsymbol{j}}}_{s}\cdot {\rm{\nabla }})\,{{\boldsymbol{n}}}_{{\boldsymbol{r}}}+\beta \,[{{\boldsymbol{n}}}_{{\boldsymbol{r}}}\times ({{\boldsymbol{j}}}_{s}\cdot {\rm{\nabla }})\,{{\boldsymbol{n}}}_{{\boldsymbol{r}}}],\end{array}$$

where *α* is the Gilbert damping constant (see also Methods). The last two terms in Eq. (2) represent the spin-transfer-torque effect due to the spin polarlized electric current: ***j***_*s*_ represents the spin current density given by the spin polarlized electric current and *β* is the coefficient of the non-adiabatic effect. We use *J* for the unit of *h*, *D*, and *K*_imp_, and 1/(*γJ*) for the unit of time *t*. The unit of the spin current density *j*_*s*_ = |***j***_*s*_| is 2*eγJ*/(*pa*^2^) for the spin polarized electric current density (*p*: polarization of magnet). (See Methods for computational details.) For the typical B20 skyrmion magnets^16^, e.g., MnSi, FeGe and so on, *J* ~ 10^−3^ eV, 1/(*γJ*) ~ 0.7 ps for $\gamma ={g}_{s}{\mu }_{B}/\hslash$ (*g*_*s*_: electron spin *g*-factor, *μ*_*B*_: Bohr magneton), and 2*eγJ*/(*pa*^2^) ~ 1.0 × 10^13^ A/m^2^ in the case that polarization of magnet *p* = 0.2 and the lattice constant *a* = 5 Å.

**Figure 1:**
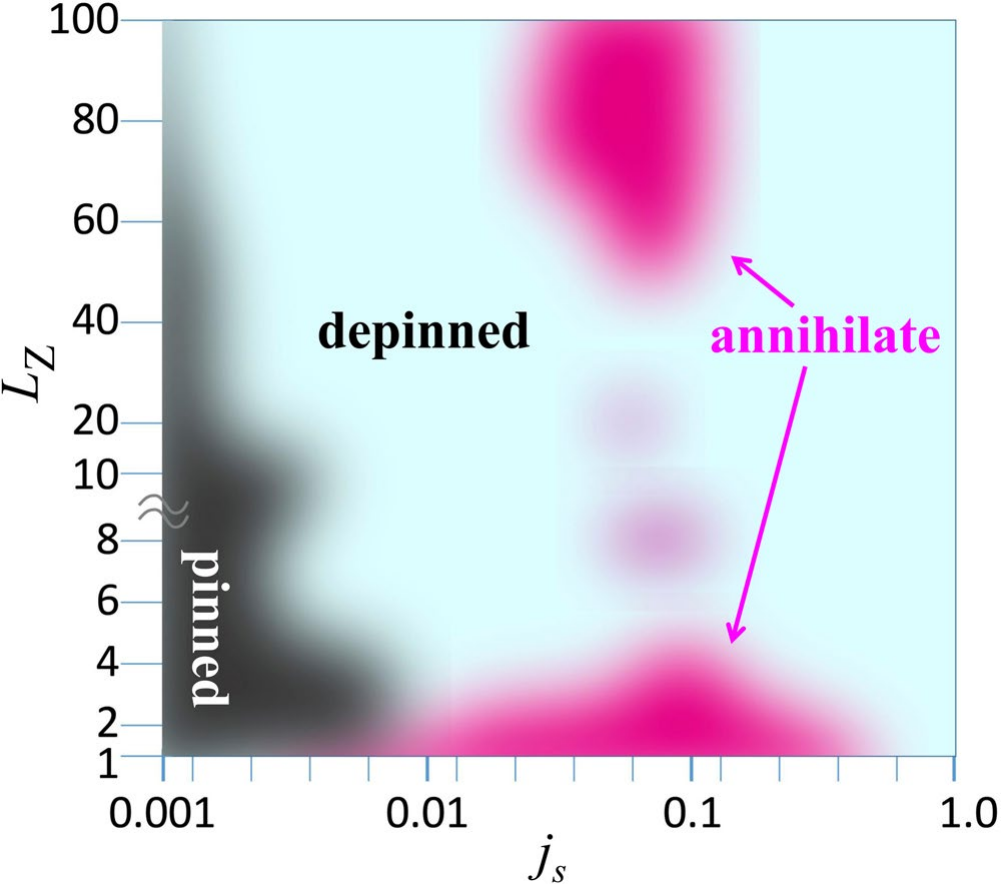
Phase diagram of current driven skyrmion string in disordered system, *L*_Z_: thickness of system, *j*_*s*_ current density to drive skyrmion string. (See Methods and Supplementary Information).

Figure 1 summarizes the dynamical phase diagram of the current-driven skyrmion string with disorder. In this study, we introduce the impurity sites with 10% concentration. Here, to examine the skyrmion-string length dependence on the current driven dynamics, we prepare system with thickness *L*_Z_ = 1 ~ 100 (see Methods). We employ the open boundary condition along *z*-direction while the periodic one for *x*- and *y*-directions.

To examine the dynamics of skyrmion string, we define the quantity,

3 $${E}_{\gamma }=\frac{1}{2\pi {L}_{Z}}\,\iiint \,{e}_{\gamma ,{\boldsymbol{r}}}\,{d}^{3}r,$$

with *γ* (=*x*, *y*, *z*) component of the emergent electric field (e-field)^47–49^

4 $${e}_{\gamma ,{\boldsymbol{r}}}=\frac{1}{2}{{\boldsymbol{n}}}_{{\boldsymbol{r}}}\cdot ({\partial }_{\gamma }{{\boldsymbol{n}}}_{{\boldsymbol{r}}}\times {\partial }_{t}{{\boldsymbol{n}}}_{{\boldsymbol{r}}}).$$

We also define the skyrmion number on the *xy*-plane at *r*_*z*_ by

5 $${N}_{sk}({r}_{z})=\frac{1}{4\pi }\,\iint \,{{\boldsymbol{n}}}_{({r}_{x},{r}_{y},{r}_{z})}\cdot ({\partial }_{x}{{\boldsymbol{n}}}_{({r}_{x},{r}_{y},{r}_{z})}\times {\partial }_{y}{{\boldsymbol{n}}}_{({r}_{x},{r}_{y},{r}_{z})})\,d{r}_{x}d{r}_{y}.$$

In the present study, for the skyrmion string without monopole/antimonopole inside system, *N*_*sk*_(*r*_*z*_) is independent of *r*_*z*_, so that we use the notation *N*_*sk*_(*r*_*z*_) = *N*_*sk*_. For such skyrmion string without distortion, it is found that

6 $${E}_{x}=-\,{N}_{sk}{v}_{d,y},\,{E}_{y}={N}_{sk}{v}_{d,x},$$

where ***v***_*d*_ = (*v*_*d*,*x*_, *v*_*d*,*y*_) is the velocity of the skyrmion string in horizontal direction. In the present case, *N*_*sk*_ = −1.

### Single-layer system (*L*_Z_ = 1)

**Figure 2:**
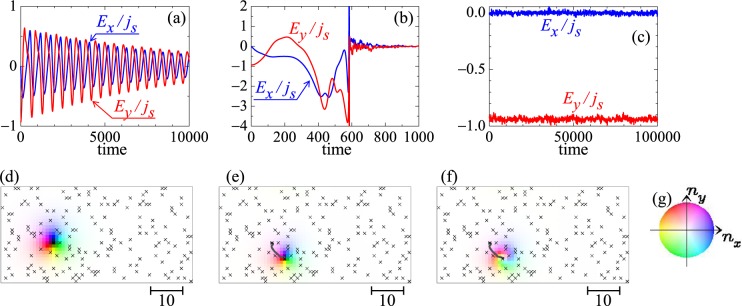
Skyrmion dynamics in single-layer system (*L*_Z_ = 1) with disorder. The time dependence of the quantities *E*_*x*_/*j*_*s*_ (blue line) and *E*_*y*_/*j*_*s*_ (red line), for *j*_*s*_ = 0.006, 0.008, and 0.8 are shown in (**a**–**c**), respectively (see text). Magnetic textures at (**d**) *t* = 0, (**e**) *t* = 578, (**f**) *t* = 586 for *j*_*s*_ = 0.008 (corresponding to the panel (b)) are shown using color code in (**g**). The brightness of the color represents the *z*-component *n*_*z*_ of the magnetic moment, i.e., black (white) is corresponding to *n*_*z*_ = −1 (*n*_*z*_ = +1). The symbol “×” represents the impurity site. In (**e** and **f**), the center-of-mass trajectory of the skyrmion is shown by solid curve (see Methods and Supplementary Information).

First, we discuss the skyrmion dynamics in *L*_Z_ = 1 system. Figure 2(a–c) show the time dependence of the quantities *E*_*x*_/*j*_*s*_ (blue line) and *E*_*y*_/*j*_*s*_ (red line), for *j*_*s*_ = 0.006, 0.008, and 0.8, respectively. The results shown in Fig. 2(a) indicate the pinning dynamics of the skyrmion, i.e., the dumped oscillation around the (meta) stable point. This is similar to the relaxation dynamics of the object with vorticity in the harmonic potential on two-dimensions. We find such pinned state of a skyrmion for *j*_*s*_ ≤ 0.006. For *j*_*s*_ = 0.008 which is larger than the critical depinning current density for this single-layer system ${j}_{s,c\mathrm{,1}}\simeq 0.006$, the skyrmion starts to move but immediately evaporates. As seen in Fig. 2(b), *E*_*x*_/*j*_*s*_ and *E*_*y*_/*j*_*s*_ have peaky structure at *t* ~ 580 by the drastic change in magnetic texture due to the skyrmion annihilation. Figure 2(d–f) show the snapshots of the magnetic texture at *t* = 0, 578, and 586, respectively. In the plot Fig. 2(c), we see ${E}_{x}/{j}_{s}\simeq 0$ and ${E}_{y}/{j}_{s}\simeq 1$ for large ${j}_{s}=0.8$. This represents that the impurity effect is reduced enough by the large *j*_*s*_. For 0.8 ≤ *j*_*s*_, the motional narrowing effect reduces the effect of impurities, and the current driven skyrmion survives.

### Multilayer systems (*L*_Z_ ≥ 2)

**Figure 3:**
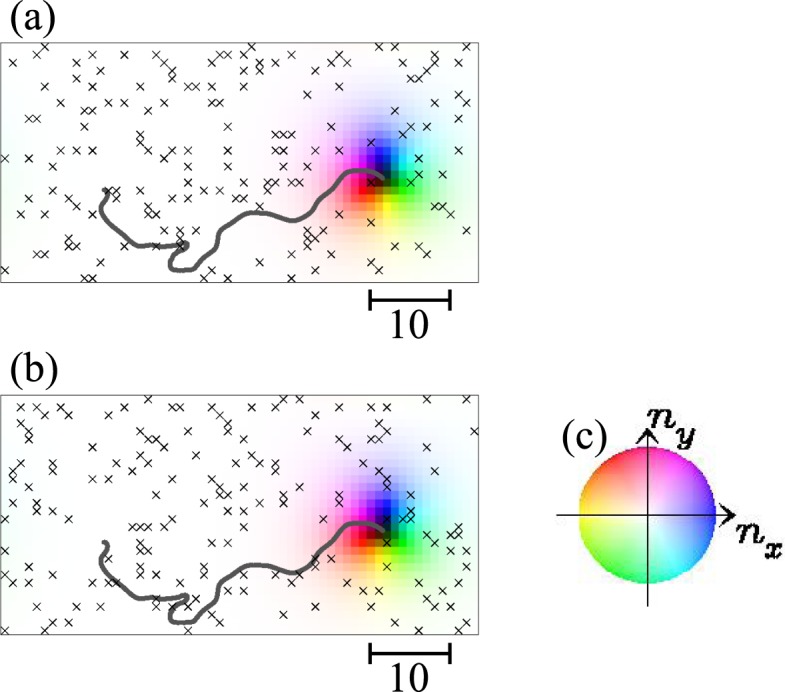
Skyrmion dinamics in two-layer system with disorder. The snapshots of the magnetic texture for *j*_*s*_ = 0.006 of the (**a**) top and (**b**) bottom layer at *t* = 5000 using color code in (**c**). In (**a** and **b**), the center-of-mass trajectory of the skyrmion is shown by solid curve (see Methods and Supplementary Information).

In *L*_Z_ = 2 system, we find the substantial suppression of the impurity effect, i.e., the reduction of the skyrmion depinning critical current density *j*_*c*_, and the range of the current density where the skyrmion annihilates is reduced (see Fig. 1). Figure 3 shows the skyrmion dynamics in two-layer system. For *j*_*s*_ = 0.006 (=*j*_*s*,*c*,1_), we see that the skyrmion is depinned and moves although the skyrmion is pinned in the single-layer system as shown in Fig. 2(a). For *j*_*s*_ < 0.004, the skyrmion is pinned as in the case of the single-layer system. In the two-layer system, we find the moving skyrmion depinned state between its pinned and annihilation regions, while this behavior is hardly seen in the single-layer system with the strong disorder as studied in the present paper.

With increasing number of stacking layer (or thickness of the system) *L*_Z_, the depinning critical current density *j*_*s*,*c*_ is further reduced and skyrmion annihilation region disappears at *L*_Z_ ~ 10. As seen in the Fig. 1, at *L*_Z_ ~ 40, we do not find skyrmion annihilation region of *j*_*s*_.

**Figure 4:**
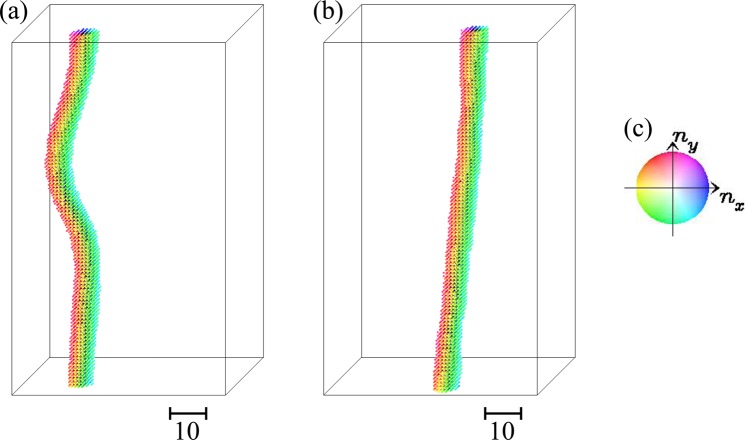
The current-driven dynamics of skyrmion string with disorder for *L*_Z_ = 100. Snapshots of the magnetic textures for (**a**) *j*_*s*_ = 0.04, (**b**) *j*_*s*_ = 1.0 are shown using color code in (**c**) for in-plane magnetic moment. To show the magnetic texture clearly, impurity sites are not indicated. The brightness of the color represents the z-component of the magnetic moment *n*_*z*_, i.e., black is corresponding to *n*_*z*_ = −1 but we do not put color for *n*_*z*_ > 0.5, i.e., the outside of the skyrmion string, to make the 3-dimensional magnetic texture clear.

**Figure 5:**
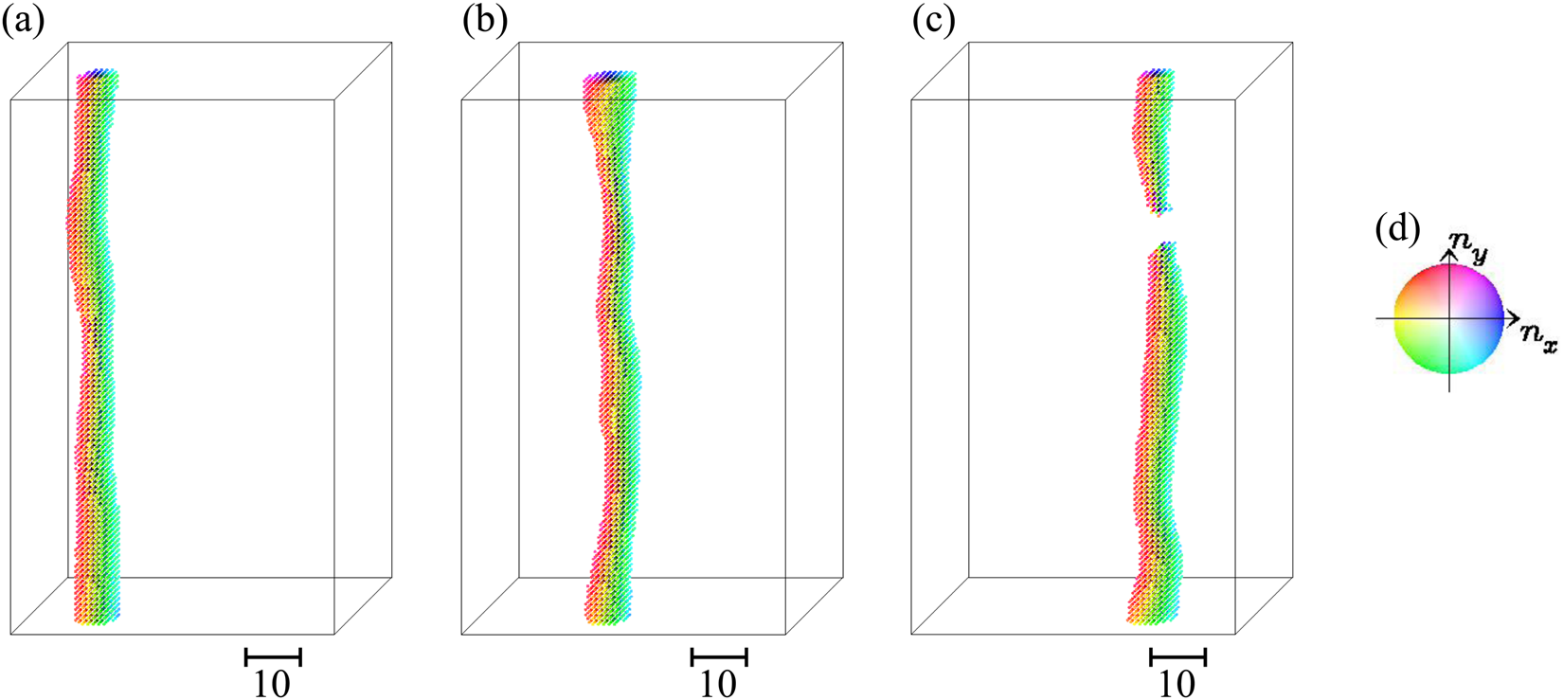
Monopole-antimonopole creation in current driven skyrmion dynamics for *L*_Z_ = 100. Magnetic textures at (**a**) *t* = 1200, (**b**) *t* = 1430, (**c**) *t* = 1740 for *j*_*s*_ = 0.06. Impurity sites are not indicated. (**d**) Color code for in-plane magnetic moment (see Methods and Supplementary Information).

**Figure 6:**
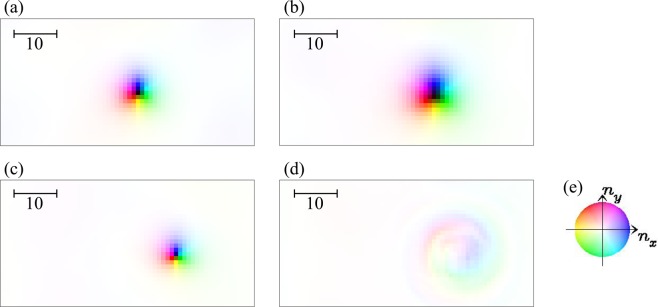
Skyrmion annihilation at the tearing point of the skyrmion string (see text). Magnetic textures in the two-dimensional cross section where the monopole-antimonopole pair-creation occurs (*r*_*z*_ = *r*_*m*-*am*_) for (**a**) *t* = 1570, (**b**) *t* = 1655, (**c**) *t* = 1730 and (**d**) *t* = 1745. Impurity sites are not indicated. (**e**) Color code for in-plane magnetic moment (see Methods and Supplementary Information).

**Figure 7:**
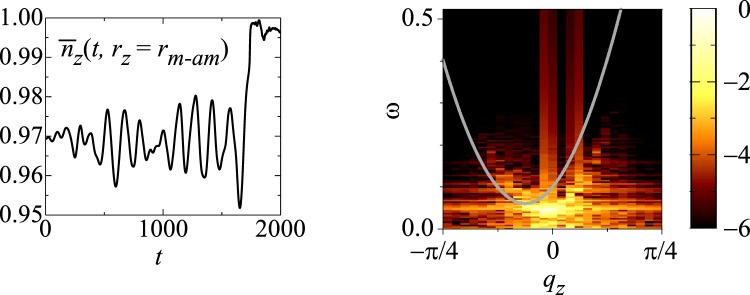
Precursor dynamics of magnetic moments toward monopole-antimonopole pair creation. (**a**) Time dependence of ${\bar{n}}_{z}$(*t*, *r*_*z*_ = *r*_*m*-*am*_) shown in Fig. 6. (**b**) Power spectra |*S*(*q*, *ω*)|^2^ for the results shown in Figs 5 and 6. The gray curve is the dispersion relation of the spin-wave for (0, 0, *q*_*z*_) with easy axis anisotropy *K*_imp_ × 0.1 for every sites (see text). To see the weak intensity clearly, contour plots of log |*S*(*q*, *ω*)|^2^ are presented.

For *L*_Z_ ≥ 60, we find that the skyrmion annihilation occurs again for 0.06 ≤ *j*_*s*_ ≤ 0.1. The dynamics without (with) skyrmion annihilation for *L*_Z_ = 100 are shown in Figs 4 and 5. Figure 5 summarizes an example of the skyrmion annihilation dynamics. In this case, the skyrmion string moves for a while, and it is broken at a point, in other words, monopole-antimonopole pair is created. The monoplole and antimonopole run through the string, and finally the (metastable) skyrmion string disappears. This skyrmion annihilation behavior is different from that in thin systems. Figure 6 shows the magnetic texture of the horizontal cross section of the system at *r*_*z*_ = *r*_*m*-*am*_ where the pair creation of monopole-antimonopole of the skyrmion string occurs. In the time evolution of the magnetic texture, Fig. 6(a–d), we find the *oscillation* of the skyrmion size. Along the current driven dynamics, the breathing mode of the skyrmion is excited due to the collision with the impurities. The characteristic collective mode propagates along the skyrmion string and shows a resonant behavior. Finally, the breathing in skyrmion size is enhanced and the part of the string is squeezed enough, so that the annihilation of the skyrmion, in other words, the monopole-antimonopole pair creation occurs (see Figs 5 and 6). The breathing dynamics of the skyrmion on the cross section of the skyrmion string at hight *r*_*z*_ is represented by the time dependence of the average of *z* component of ***n***_***r***_, i.e.,

7 $${\bar{n}}_{z}(t,{r}_{z})=\frac{1}{{L}_{{\rm{X}}}{L}_{{\rm{Y}}}}\,\iint \,{n}_{z,({r}_{x},{r}_{y},{r}_{z})}d{r}_{x}d{r}_{y},$$

where *L*_X_ × *L*_Y_ is the horizontal cross-sectional area of the system. Figure 7(a) shows ${\bar{n}}_{z}$(*t*, *r*_*z*_ = *r*_*m*-*am*_) for the results shown in Fig. 6: At around $t\simeq 1650,$  ${\bar{n}}_{z}$(*t*, *r*_*z*_ = *r*_*m*-*am*_) drops and reaches smallest value during this dynamics. After that, in the time duration 1650 ≤ *t* ≤ 1750, ${\bar{n}}_{z}$(*t*, *r*_*z*_ = *r*_*m*-*am*_) grows rapidly and saturates → 1. This represents the overshoot behavior of the skyrmion breathing dynamics, i.e., the skyrmion is expanding largely first, and later it is squeezing enough to annihilate the skyrmion. On the skyrmion dynamics, the spin wave excitations^50,51^ on the skyrmion string play important role. Figure 7(b) shows the power spectrum |*S*(*q*, *ω*)|^2^ defined by

8 $$S(q,\omega )=\frac{1}{T}\frac{1}{{L}_{Z}}\,{\int }_{0}^{T}\,dt\,\int \,{e}^{-iq{r}_{z}+i\omega t}{\bar{n}}_{z}(t,{r}_{z})d{r}_{z},$$

for the time duration *T* (=1600 is used for Fig. 7(b)) before the monopole-antimonopole pair creation. This represents the collective mode dynamics along skyrmion string towards the monopole-antimonopole creation. The gray curve in Fig. 7(b) indicates the spin-wave dispersion relation of this system,

9 $$\begin{array}{ccc}\omega ({\boldsymbol{q}}) & = & h+2K\,+\,6J[1-\frac{1}{3}(\cos \,{q}_{x}+\,\cos \,{q}_{y}+\,\cos \,{q}_{z})]\,+\,2D\,\sin \,{q}_{z},\end{array}$$

for (0, 0, *q*_*z*_) but an easy-axis anisotropy *K* = *K*_imp_ × 0.1 is applied for every sites. Because of the DM interaction ***D***_Z_ (=$D\hat{{\boldsymbol{z}}}$) between the sites along $\hat{{\boldsymbol{z}}}$ direction, the spin-wave excitation is not symmetric in *q*_*z*_ axis. It is indicated that the breathing collective mode is below the continuum of the spin waves for the positive *q*_*z*_ while it is buried in the negative direction. This results in the large directional propagation of the breathing mode.

For 0.1 < *j*_*s*_, we do not see the skyrmion annihilation, i.e., the spin-transfer-torque effect by *j* is large enough compare to the impurity effect. Therefore, the moving skyrmion string becomes rather straight compare to that for weak *j*_*s*_ < 0.06 (see also Fig. 4(a,b)).

The impurity sites are given by the random number, so that the impurity configuration is layer dependent. This layer dependence of the impurity configuration affects the impurity effect. In the system with layer *independent* impurity configuration, skyrmion dynamics with ***D***_Z_ = **0** is the same as that in single-layer system.

**Figure 8:**
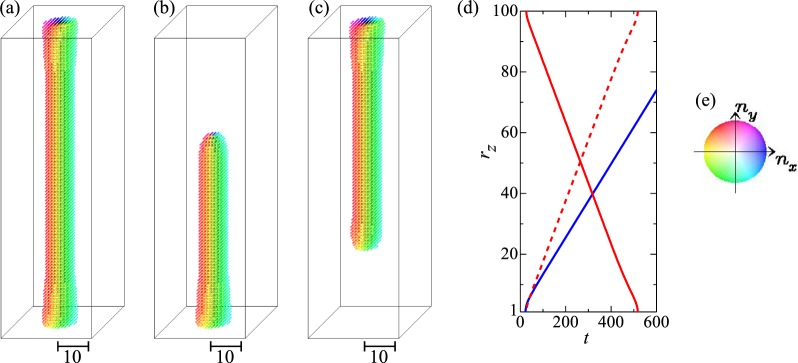
Monopole and antimonopole dynamics without impurities for *L*_Z_ = 100 (see text). For the Gilbert damping constant, *α* = 1.0 is used. (**a**) Initial state at *t* = 0. For this magnetic texture, a strong *h* = 1.0 is applied only on top or bottom surface for *t* ≥ 0. (**b**) Magnetic texture at *t* = 400. (**c**) The same as (**b**) but the strong *h* = 1.0 is applied at bottom surface. (**d**) Monopole (red solid line) and antimonopole (blue line) position in *z* axis as functions of time *t*. The broken red line is the flipped red solid line in vertical direction to see the difference of the monopole velocity and antimonopole one. See also Methods. (**e**) Color code for in-plane magnetic moment used in (**a**–**c**).

As discussed above, the annihilation of the skyrmion string is achieved by the monopole and antimonopole motion along the string. We now consider the propagation of monopole and antimonopole along the skyrmion string. Figure 8 shows the monopole and antimonopole motion in the system without impurity *K*_imp_ and current *j*_*s*_ (see also Methods). Figure 8(a) shows the initial state, i.e., the (metastable) skyrmion string. At the top surface, we apply strong *h* = 1.0 for *t* ≥ 0 to annihilate the skyrmion only on the top surface. By this procedure, the monopole is created, which begins to move downward in the system, since the skyrmion is the metastable state in this parameter set. Figure 8(b) shows the snapshot of the magnetic texture at *t* = 400. Here, we call monopole (antimonopole) for the magnetic texture with positive (negative) div***n***_***r***_ inside the system. In the same way, we apply strong *h* = 1.0 on the bottom surface and examine the antimonopole dynamics (see Fig. 8(c)). The monopole (antimonopole) runs through the skyrmion string, and finally the string totally disappears. Figure 8(d) shows the monopole (antimonopole) position as a function of time (see also Methods). To compare the monopole velocity and the antimonopole one, the red broken line is also presented in Fig. 8(d).

**Figure 9:**
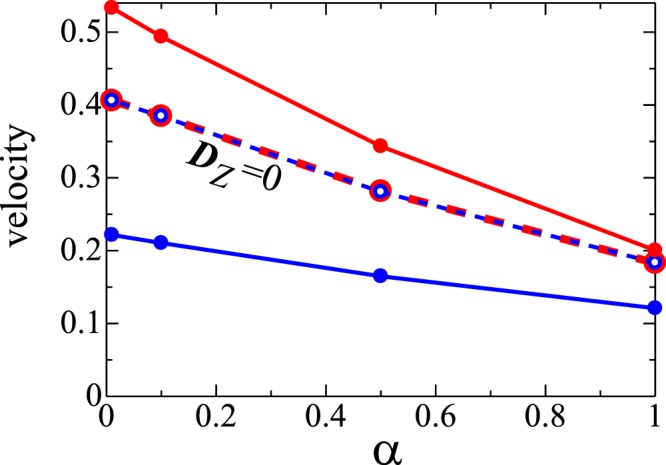
Monopole (antimonopole) velocity as a function of *α*. Red (blue) line with solid dot represents the monopole (antimonopole) velocity for $\{{{\boldsymbol{D}}}_{{\rm{X}}}=D\hat{{\boldsymbol{x}}},{{\boldsymbol{D}}}_{{\rm{Y}}}=D\hat{{\boldsymbol{y}}},{{\boldsymbol{D}}}_{{\rm{Z}}}=D\hat{{\boldsymbol{z}}}\}$. Broken line with open dot is the case for ***D***_Z_ = **0**.

We examine the *α* dependence of the monopole and antimonopole motion (see Fig. 9). With decreasing *α*, the velocity of the monopole/antimonopole is enhanced. This large directional dependence of the (anti)monopole motion will lead to the large nonreciprocal responses of the system, which is left for future studies. We also examine the case with ***D***_Z_ = **0** where the directional dependence disappears as expected.

## Discussion

It has been experimentally shown^52,53^ that the skyrmions in three-dimensional bulk form samples are driven by electric current with current density ~10^6^ A/m^2^ being orders of magnitude smaller than that for magnetic domain wall motion. This was assumed to be an advantageous for technological application in the earlier studies^16,54^. However, it is reported^55^ that the microscale-fabricated MnSi sample shows very large threshold current density ~10^8^ A/m^2^ which is two orders magnitude larger than that in bulk samples. The experimental studies for the manipulation of the skyrmion have been extensively developed after the discovery of the room-temperature skyrmion induced by interface DM interaction in artificial thin-film heterostructures^20–28^. Here, the spintronics friendly centrosymmetric materials are used, where ***D***_X_ and ***D***_Y_ are induced by the interface^56^ while ***D***_Z_ = 0. In those systems, the threshold current density to drive skyrmion(s) is ~10^10^–10^11^ A/m^2^ and is 4 or 5 orders of magnitude larger than that of conventional bulk skyrmion magnets. In view of present numerical simulation study for the current driven skyrmion, the threshold current density is not only determined by impurity strength and concentration but also by thickness of the system, i.e., with decreasing thickness of the system, the threshold current density is several orders of magnitude enhanced. In addition to this, such *two-dimensionality* is rather enhanced in the thin system with ***D***_Z_ = **0** where the twist of magnetic moments in *z* direction by DM interaction is missing. For longer skyrmion string, although the threshold current density is reduced, the monopole-antimonopole creation instability appears. In other words, for the stability of the skyrmion, there exists optimum sample-thickness which is typically ~100 nm for lattice constant *a*~5Å.

In our numerical study, for the metastable skyrmion under the strong magnetic field *h*, the velocity of monopole/antimonopole (along skyrmion string) is estimated to be ~10^2^ m/sec for typical parameters (see the lines below Eq. (2)) of bulk skyrmion materials. The velocity is not much faster than the velocity of current driven skyrmion(s) and is also getting slow for smaller *h*. Our numerical study also suggests that in bulk samples, the impurity effect on current driven skyrmion is suppressed and skyrmion annihilation instability with monopole/antimonopole creation appears. The monopole/antimonopole motion causes emergent e-field. The experiments (e.g. ref. ^55^) to measure the characteristics of e-field responsible for the skyrmion string dynamics will also be useful to observe the monopole/antimonopole dynamics. Such experiments are desired.

## Conclusion

We numerically studied the current driven dynamics of a magnetic skyrmion string in the film form system in the presence of pinning effect. It was shown that the pinning effect is strongly enhanced for shorter skyrmion string which is corresponding to the skyrmion dynamics in thinner samples. For longer skyrmion string, it was also found that the skyrmion string annihilation instability due to the monopole-antimonopole pair creation. For the instability, it was found that the collective breathing mode on the skyrmion string plays a crucial role. The theoretical results suggest that there is an optimum sample thickness for the stability of the current driven skyrmion string.

## Methods

For the LLG simulation of the single skyrmion string dynamics, we use 60 × 30 × *L*_Z_ (*L*_Z_ = 1 ~ 100) finite size system with periodic (open) boundary condition in horizontal (vertical) direction. For the initial state, we first prepare the metastable at *K*_imp_ = 0, and obtain the relaxed state. To obtain the phase diagram Fig. 1, we examine the skyrmion dynamics for {*j* = 0.001. 0.002, 0.004. 0.006, 0.008, 0.01, 0.02, 0.04, 0.06, 0.08, 0.1, 0.2, 0.4, 0.6, 0.8, 1.0}, and the number of layers are 1, 2, 4, 6, 8, 10, 20, 40, 60, 80 and 100. Most calculations are done within a time duration 200000.

The numerical condition *α* = *β* = 0.01 is used for the numerical simulations except for the results summarized in Figs 8 and 9. In the condition *α* = *β*, the Hall angle of current driven skyrmion is zero for *K*_imp _= 0, so that we can examine purely impurity driven scattering effect.

For the impurity sites ${{\boldsymbol{r}}}_{{\rm{i}}}\in {\rm{\Lambda }}$ (${\rm{\Lambda }}$: set of the random sites), we use the random-number generator developed by M. Matsumoto and T. Nishimura (http://www.math.sci.hiroshima-u.ac.jp/~m-mat/MT/emt.html).

The center-of-mass of the skyrmion at *r*_*z*_ is calculated by $\tfrac{{\sum }_{{r}_{x},{r}_{y}}\,[(\,-\,{N}_{sk}({r}_{z})\times {n}_{z,({r}_{x},{r}_{y},{r}_{z})}-1)\times ({r}_{x},{r}_{y},{r}_{z})]}{{\sum }_{{r}_{x},{r}_{y}}\,(\,-\,{N}_{sk}({r}_{z})\times {n}_{z,({r}_{x},{r}_{y},{r}_{z})}-1)}$, and plot the trajectory in Figs 2 and 3.

The discontinuity of *N*_*sk*_(*r*_*z*_) as function of *t* and *r*_*z*_ is used to identify the broken point of the skyrmion string *r*_*m*-*am*_ and monopole/antimonopole position in *z* axis.

## Supplementary information

Supplementary Information

Movie for Fig.2.

Movie for Fig.3(a).

Movie for Fig.3(b).

Movie for Fig.5.

Movie for Fig.6.
