# Supplementary material for: Dynamics of skyrmion in disordered chiral magnet of thin film form
Source: Sci Rep. 2019 Mar 25;9:5111. doi: 10.1038/s41598-019-41441-5 (PMC6434043; doi:10.1038/s41598-019-41441-5)
Supplement: Supplementary file 1 — Supplementary Information [file 41598_2019_41441_MOESM1_ESM.pdf]

Dynamics of skyrmion in disordered chiral magnet of thin film form

Supplementary Information

Wataru Koshibae, Naoto Nagaosa

Supplementary figure for Fig.1

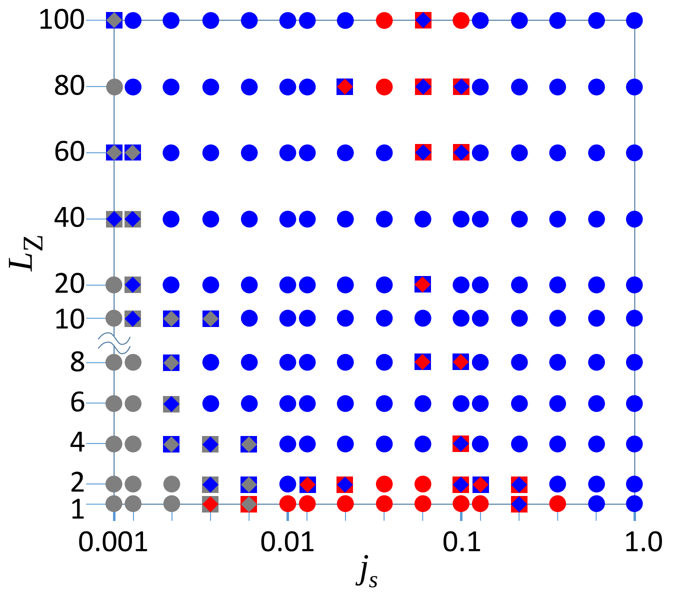

Numerical simulation data for the dynamical phase diagram shown in Fig. 1. The vertical axis L_Z and horizontal axis j_s stand for the system thickness and driving current density, respectively. The meaning of the symbol color is summarized in the following, gray: pinned state is achieved at the final state, red: skyrmion annihilation occurs, blue: current driven skyrmion is moving without pinning and annihilation behavior. At each point (L_T, j_s), we try three different impurity spatial configurations. In this try, at the symbols, circle, square, diamond, the current driven skyrmion shows the final state represented by the symbol color three times, two times, and once, respectively, (e.g., at the red symbols ●, ■, ◆, skyrmion annihilation is found three times, two times and once, respectively.)

Supplementary Movies

Fig2.avi: Movie for Fig.2.

The skyrmion dynamics in single-layer system (L_Z= 1). The parameter set {J=1.0, D=0.2, K_{imp}=0.2, h=0.06, α=β=0.01, j_s=0.008} is used. The impurity site is represented by the symbol ×.

Fig3a.avi: Movie for Fig.3(a).

The skyrmion dynamics in two-layer system ($L_Z = 2$). The top layer magnetic texture is shown. The parameter j_s=0.006 is used, and other parameters are the same as those used for Fig.2.

Fig3b.avi: Movie for Fig.3(b).

The same as Fig3a.avi, but the bottom layer magnetic texture is shown.

Fig5.avi: Movie for Fig.5.

The skyrmion annihilation dynamics by monopole-antimonopole pair creation for L_Z=100. The parameter j_s=0.06 is used, and other parameters are the same as those used for Fig.2. To show the magnetic texture clear, the impurity sites are not indicated.

Fig6.avi: Movie for Fig.6.

The magnetic texture on the horizontal cross section at the tearing point of the skyrmion string shown in Fig5.avi. To show the magnetic texture clear, the impurity sites are not indicated.
